# Sustainable Collagen Composites with Graphene Oxide for Bending Resistive Sensing

**DOI:** 10.3390/polym15193855

**Published:** 2023-09-22

**Authors:** Mireia Andonegi, Daniela M. Correia, Nelson Pereira, Carlos M. Costa, Senentxu Lanceros-Mendez, Koro de la Caba, Pedro Guerrero

**Affiliations:** 1 BIOMAT Research Group, Escuela de Ingeniería de Gipuzkoa, University of the Basque Country (UPV/EHU), Plaza de Europa 1, 20018 Donostia-San Sebastián, Spain; mireia.andonegui@ehu.eus (M.A.); pedromanuel.guerrero@ehu.es (P.G.); 2 Centre of Chemistry, University of Minho, 4710-057 Braga, Portugal; danielamscorreia@gmail.com; 3 Physics Centre of Minho and Porto Universities (CF-UM-UP), University of Minho, 4710-057 Braga, Portugal; nelsonpereira@fisica.uminho.pt (N.P.); cmscosta@fisica.uminho.pt (C.M.C.); 4 Laboratory of Physics for Materials and Emergent Technologies (LapMET), University of Minho, 4710-057 Braga, Portugal; 5 Institute of Science and Innovation for Bio-Sustainability (IB-S), University of Minho, 4710-053 Braga, Portugal; 6 BCMaterials, Basque Center for Materials, Applications and Nanostructures, UPV/EHU Science Park, 48940 Leioa, Spain; 7 Ikerbasque, Basque Foundation for Science, 48009 Bilbao, Spain; 8 Proteinmat Materials SL, Avenida de Tolosa 72, 20018 Donostia-San Sebastián, Spain

**Keywords:** sustainability, smart materials, biocomposite, sensor

## Abstract

This work reports on the development of collagen films with graphene oxide nanoparticles (GO NPs), aiming toward the development of a new generation of functional sustainable sensors. For this purpose, different GO NP contents up to 3 wt % were incorporated into a collagen matrix, and morphological, thermal, mechanical and electrical properties were evaluated. Independently of the GO NP content, all films display an increase in thermal stability as a result of the increase in the structural order of collagen, as revealed by XRD analysis. Further, the inclusion of GO NPs into collagen promotes an increase in the intensity of oxygen characteristic absorption bands in FTIR spectra, due to the abundant oxygen-containing functional groups, which lead to an increase in the hydrophilic character of the surface. GO NPs also influence the mechanical properties of the composites, increasing the tensile strength from 33.2 ± 2.4 MPa (collagen) to 44.1 ± 1.0 MPa (collagen with 3 wt % GO NPs). Finally, the electrical conductivity also increases slightly with GO NP content, allowing the development of resistive bending sensors.

## 1. Introduction

In the last two decades, biopolymers have emerged as promising candidates for the development of sustainable solutions in areas ranging from food packaging and biomedicine to energy storage and electronics in order to meet the growing need of advanced materials in the scope of the principles of Green Chemistry and the 2030 Agenda for Sustainable Development [1,2,3]. In particular, the valorization of naturally abundant and renewable raw materials, such as polysaccharides and proteins, using environmentally friendly strategies has become a promising opportunity not only to reduce the use of petroleum-derived polymers but also to minimize waste generation and produce high value-added products [4,5]. In this context, collagen represents a potential candidate for a wide variety of functional applications thanks to its structural and biological properties, combined with its ease of extraction and the possibilities for large-scale production [6]. Collagen is the most abundant protein in vertebrates, and it can be extracted from almost every vertebrate, including fish (scales, bones, swimming bubbles and skin); nonetheless, bovine, porcine and chicken skin and bones are the main sources at the commercial scale [7,8]. Furthermore, its triple helix structure forms insoluble fibers, providing collagen with a high integrity, inherent water stability and mechanical tensile strength [9]. Its high flexibility and stretchability makes collagen an attractive candidate for the development of sustainable sensing devices, including bendable, conformable and wearable sensors [10,11]. Additionally, the combination of collagen and other biopolymers with inorganic fillers has emerged as a promising route to obtain sustainable bionanocomposites with added functionalities that meet the current and future needs of modern technology [4,12]. In particular, several studies have reported the use of 1D and 2D carbon monolayer-based nanomaterials (carbon nanotubes, graphene and graphene oxide (GO)) as fillers in different biomaterial matrices in order to fabricate devices for various applications, ranging from wearable biosensors to functional implants and body protection [13,14]. The incorporation of those carbon-based nanomaterials within the biopolymer matrix provides mechanical reinforcement and functional properties, such as electrical and thermal conductivity, gas barrier properties and sensing abilities [15].

Among carbon monolayer-based nanoparticles, GO has become one of the most widely researched materials, based on the cost-effective and large-scale production of graphene-based materials [13,16]. GO is an oxide derivative of graphene composed by a graphene sheet with carboxylic groups at its edges and hydroxyl and epoxide groups on its basal plane [17]. The presence of oxygen on the edges and basal planes of GO increases its hydrophilicity and enhances its water dispensability in comparison with graphene, and it Is non-toxic and biocompatible at low concentrations [18]. Furthermore, the unique properties of GO, such as high carrier mobility at room temperature, a large specific surface area, high mechanical strength, thermal conductivity, functionalized surfaces, strong UV absorption and fluorescence, makes it one of the most promising materials for biosensors, therapeutics and tissue engineering, as well as electronics [12]. Furthermore, the scalability of production and convenient processing of GO has led to its emergence as an important precursor for the fabrication of transparent conductive films [19].

Several studies have reported the combination of collagen with GO in dentistry and tissue regeneration [18,20]. GO improves the physical properties of collagen, maintaining its biocompatibility and non-cytotoxicity due to the interfacial interactions between the functional groups on GO and the collagen matrix, which enable the stress transfer from collagen to GO. Panzavolta et al. (2014) reported a 50% increment in Young’s modulus and >60% in fracture stress by adding only 1% of GO to a collagen matrix [21]. Furthermore, the addition of GO increases the stability of collagen chains and hinders their denaturation [22].

However, multifunctional sensors based on collagen nanocomposites with graphene oxide are still poorly explored. Taking into account the potential application of the sensors developed for biomedical applications, collagen was selected due to its characteristics, especially its biocompatibility and its capacity to mimic the extracellular matrix, contributing to the progressive transition from synthetic to natural polymers that is mandatory to ensure a sustainable future. In this context, this work focuses on the production of collagen composites with different contents of GO nanoparticles (GO NPs) to be applied as a resistive bending sensor. These composites were prepared through solution casting by varying the amount of GO NPs. The thermal, physicochemical, morphological, mechanical and electrical properties of the composites were analyzed. In addition, a resistive bending sensor was developed to demonstrate the suitability of the developed composites for a new generation of sustainable sensors.

## 2. Materials and Methods

### 2.1. Materials

Bovine collagen was supplied by Proteinmat SL (Donostia, Spain); graphene oxide nanoparticles (GO NPs), with a surface area of 450–500 m^2^ g^−1^, apparent density of 6.5 g cm^−3^, electrical conductivity of 1.2 S cm^−1^ and C/O ratio of 55.8, were provided by Abalonyx AS; and acetic acid by Panreac Quimica S.L.U (Barcelona, Spain).

### 2.2. Sample Preparation

Collagen films with different GO NP contents (0, 0.25, 0.50, 0.75, 1 and 3 wt %) were prepared by solution casting. Collagen and the amount of GO NPs required for each formulation were incorporated into 0.5 M acetic acid (1:40 collagen/acetic acid). The mixtures were maintained at room temperature under continuous stirring at 150 rpm for 2 h and then poured into Petri dishes and left to dry at room temperature for 5 days to obtain the films. The films were designated as 0.25GO, 0.50GO, 0.75GO, 1GO and 3GO, thus indicating the GO NP content. Films without GO NPs were considered as control films. All films were conditioned in a climatic chamber (Alava Ingenieros, Madrid, Spain) at 25 °C and 50% relative humidity before testing. Samples with an average thickness of ~100 μm were obtained independently of the filler content.

### 2.3. Sample Characterization

Differential scanning calorimetry (DSC) was carried out in a Mettler Toledo DSC 822. Samples (3.0 ± 0.2 mg) were sealed in aluminum pans to avoid mass loss during the experiment. Filled pans were heated from 25 to 250 °C at a rate of 10 °C/min under inert atmosphere conditions (10 mL N_2_/min) to avoid thermo-oxidative reactions.

Fourier transform infrared (FTIR) spectroscopy with attenuated total reflectance (ATR) mode was performed by using an equipped Alpha II Compact FTIR spectrometer. A total of 32 scans were carried out from 4000 to 800 cm^−1^ at 4 cm^−1^ resolution.

X-ray diffraction (XRD) measurements were performed at 40 kV and 40 mA with a diffraction unit (PANalytical Xpert PRO, Madrid, Spain), generating the radiation from a Cu-Kα (λ = 1.5418 Å) source. Data were recorded from 2 to 50°.

Before performing scanning electron microscopy (SEM) analysis, the films were placed on a metal stub and coated with gold using a JEOL fine-coat ion sputter JFC-1100 and argon atmosphere. Samples were observed using a Hitachi S-4800 scanning electron microscope (Hitachi, Madrid, Spain) at 15 kV accelerating voltage.

X-ray photoelectron spectroscopy (XPS) was performed in a SPECS spectrometer using a monochromatic radiation equipped with Al Kα (1486.6 eV). The binding energy was calibrated by Ag 3d5/2 peak at 368.28 eV. All spectra were recorded at a 90 ° take-off angle. Survey spectra were recorded with 1.0 eV step and 40 eV analyzer pass energy and the high-resolution regions with 0.1 eV step and 20 eV pass energy. All core level spectra were referenced to the C 1s neutral carbon peak at 284.6 eV. Spectra were analyzed using the CasaXPS 2.3.19PR1.0 software, and peak areas were quantified with a Gaussian–Lorentzian fitting procedure.

Bone-shaped samples (4.75 mm × 22.25 mm) were cut and an Instron 5967 mechanical testing system (Instron, Barcelona, Spain) was used to carry out tensile tests at 1 mm/min, according to the ASTM D 638-03 standard [23]. Analysis of variance (ANOVA) was carried out with SPSS software (SPSS Statistic 25) to determine significant differences between samples. Tukey’s test with a statistical significance at the *p* < 0.05 level was considered for multiple comparisons among different systems.

The electrical conductivity (*σ*) of the GO/collagen composites was obtained at room temperature by applying a voltage between ±10 V and reading the resulting current using a Keithley 287 picoammeter/voltage source. Measurements were performed along the thickness of the samples in a parallel plate configuration. The electrical conductivity (*σ*) value was obtained through Equation (1):

(1) $$\sigma =\frac{d}{R\cdot A}$$

where *d* is the sample thickness, *R* is the composite electrical resistance, evaluated through the slope of the I-V curves, and A is the electrode area (5 mm^2^).

### 2.4. Bending Sensor Developed

**Figure 1:**
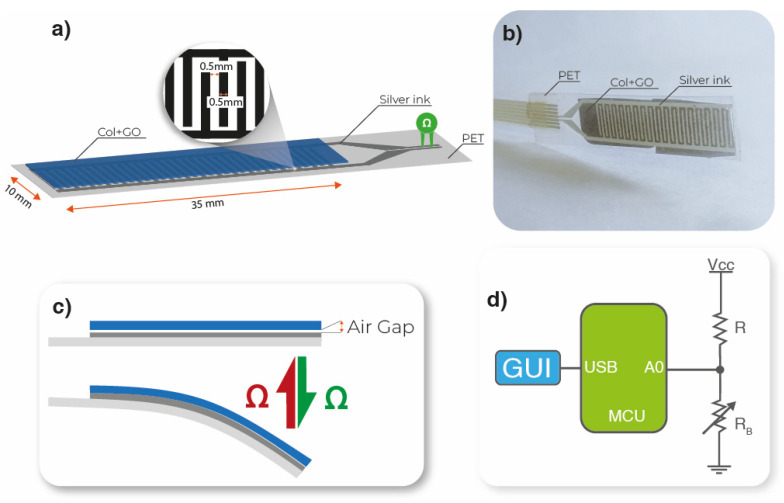
(**a**) Schematic representation and (**b**) photograph of the bending sensor. (**c**) Schematic representation of the bending mechanism. (**d**) Schematic representation of the acquisition electronic circuit.

An interdigit (35 mm × 10 mm) with a channel of 0.5 mm width was screen-printed on top of a PET substrate using a screen-printing technique. A mesh of 100 threads/cm was used in a manual screen-printing machine. The electrodes were deposited with silver nanoparticle ink (Novacentrix Metalon HPS-021LV). The ink was cured on an electrical convection oven at 80 °C for 1 h. GO/collagen films were placed and glued with transparent film tape on top of the interdigit electrode, allowing a small air gap between the two materials, as shown in Figure 1a–c.

The developed bending sensors were connected to a voltage divider (where the R_B_ resistance variation is converted to a voltage variation R = 10 MΩ), followed by an analog-to-digital converter (A0) (voltage to digital conversion) present in the microcontroller Arduino Uno (MCU). The converted data were transferred by a universal serial bus (USB) to a computer for data visualization in a graphical user interface (GUI), as illustrated in Figure 1d.

## 3. Results and Discussion

### 3.1. Thermal and Physicochemical Properties

**Figure 2:**
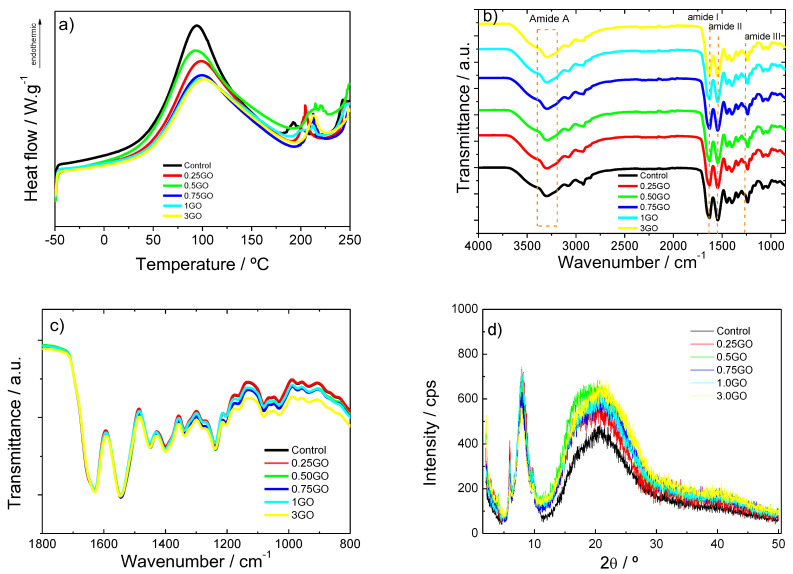
(**a**) DSC curve, (**b**,**c**) FTIR spectra from 4000 to 800 cm^−1^ and 1800 to 800 cm^−1^, respectively, and (**d**) XRD patterns of collagen films with different contents of GO NPs.

**Figure 3:**
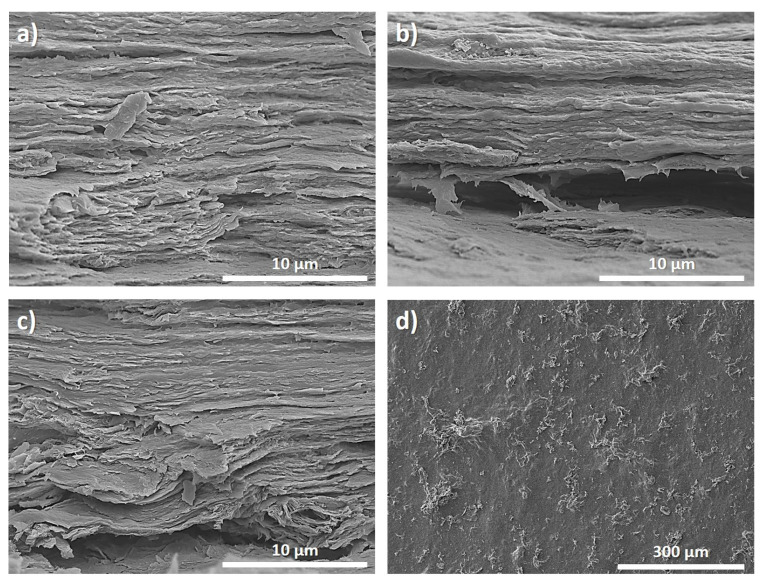
SEM images of (**a**) control (pristine collagen), (**b**) 1GO and (**c**) 3GO film cross-sections. (**d**) Surface SEM image of 3GO film.

The thermal stability of collagen films and collagen films incorporating GO NPs were evaluated by DSC analysis. As shown in Figure 2a, all samples showed an endothermic peak between 35 °C and 180 °C related to dehydration and the thermal denaturation of the amorphous region of collagen, respectively [24]. The effect of GO NPs on the glass transition temperature (T_g_), thermal denaturation temperature (T_d_) and enthalpy (ΔH) are shown in Table 1. It is observed that the T_g_ and T_d_ values increased from 46.9 °C and 94.2 °C to 48.2 °C and 99.4 °C, respectively, when 0.25 wt % GO NPs were added compared to the control film. For films with higher NP contents, it did not cause a greater temperature increase. These results show that the addition of GO NPs affects the T_d_ value, which may be due to the increase of the structural order observed by XRD analysis (Figure 3). Concerning ΔH, which represents the energy required for the release of free and bound water and the denaturation of collagen [25], this value decreased progressively from 275.7 mJ/g to 219.7 mJ/g with the addition of GO NPs. This decrease may be due to the changes of the collagen structure, as shown in Figure 2d by XRD analysis. This result could also be related to interactions between collagen and GO.

In order to assess the interactions among collagen and GO, FTIR analysis was carried out and the resulting spectra of the films are shown in Figure 2b,c. All samples showed the characteristic absorption bands of proteins: amide A at ~3300 cm^−1^ (N–H and O–H stretching), amide I at ~1630 cm^−1^ (C=O stretching), amide II at ~1542 cm^−1^ (N–H bending) and amide III at ~1240 cm^−1^ (C–N stretching) [26,27]. With the addition of GO NPs, the intensity of the broad band at 3300 cm^−1^ and that of the band at 1049 cm^−1^ increased, since the vibration bands of GO related to OH and C–O groups appear typically at 1050 and 3400 cm^−1^, respectively [28,29]. These results indicate that the abundant oxygen-containing functional groups present in the GO NPs’ surface can provide interactions with collagen amino groups [30].

### 3.2. Morphological and Surface Analyses

In order to relate the abovementioned properties with the structure of collagen films and GO NPs, SEM, XRD and XPS analyses were carried out. All samples showed similar semi-crystalline XRD patterns (Figure 2d), with a broad peak at 20°, associated with the diffuse scattering of collagen fibers, and a peak at 7° that represents the triple helix structure of collagen, related to its crystalline structure [31,32].

The structural order of collagen increased when GO NPs were added, regardless of GO NP content, as shown by the increase of the band at 20°, implying an amorphization due to the interaction of collagen with GO NPs. This increase may be due to the GO-collagen physical interactions of the hydroxyl and carboxylic groups in GO NPs with the amino groups in collagen due to the abundant oxygen-containing groups on the GO NPs’ surface [30]. Furthermore, the increase of GO NP content did not affect the triple helix structure of collagen since the intensity of the peak at 7° is similar in all samples. It should be noted that no peaks corresponding to the crystal structure of GO NPs were found, indicating that GO NPs were homogenously dispersed in the collagen matrix [31].

Regarding the film microstructure (Figure 3), SEM images of the film cross-section showed the fibrillar structure of collagen, and no GO NP agglomeration was observed independently of the filler content (Figure 3a–c), confirming the good dispersion of GO NPs in the collagen matrix [33,34], as also indicated by XRD analysis. Furthermore, the dispersion of GO is observed in Figure 3d for films with higher GO content. From Figure 3d, a good dispersion and excellent compatibility between the filler and the collagen matrix is observed.

**Figure 4:**
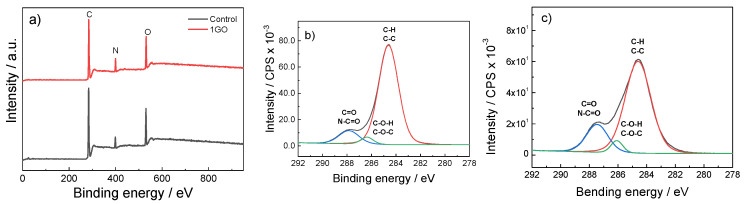
XPS survey spectra of (**a**) control and 1GO films and deconvolution curves of C 1s features for (**b**) control and (**c**) 1GO films.

XPS was performed to obtain a detailed view of the elemental composition of the film surface. As can be observed in Figure 4a, the predominant peaks, identified at 284.6 eV, 399.1 eV and 531.1 eV, are related to C 1s, N 1s and O 1s, respectively [35]. Moreover, the C 1s can be deconvoluted into three peaks, attributed to the aliphatic carbons (284.0 eV; C–H/C–C), carbons associated with oxygen or nitrogen atoms (286.1 eV; C–O/C–N) and carbons in the collagen peptide chain (287.5 eV; C=O/N–C=O) [36]. The relative areas of these three peaks changed from the control (Figure 4b) to 1GO (Figure 4c) films.

A quantitative analysis of the areas under the curves was performed. As presented in Table 2, the peak area corresponding to C–H/C–C bonds decreased, while those related to C–O/C–N and C=O/N–C=O increased, as well as those related to the C=N/C–N and O–C=O/O=C–N groups, indicating the migration of GO sheets to the hydrogel/air interface, and thus the increase in the hydrophilic character of the surface.

### 3.3. Mechanical Properties

Tensile tests were performed in order to evaluate the effect of GO NPs on the mechanical behavior of the composites. The GO-collagen interface plays an important role in the interactions between the filler and the matrix and, thus, in the interface adhesion and in the mechanical properties of composite films [37]. As can be seen in Table 3, tensile strength (TS) increased (*p* < 0.05) when GO NPs were incorporated into collagen formulations. This increase is the result of the physical interactions between collagen and GO NPs as well as of the increase in the structural order of collagen observed by XRD. Concerning elongation at break (EAB), no significant change (*p* > 0.05) was observed up to 1 wt % GO NPs, when a slight decrease in EAB values was observed, attributed to the rigid structure of GO [38,39].

### 3.4. Electrical Properties

**Figure 5:**
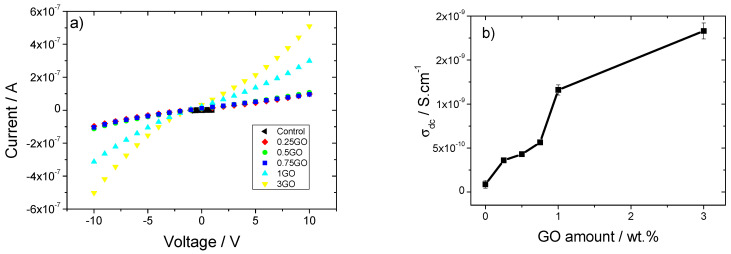
(**a**) Current-voltage (I-V) curves and (**b**) electrical conductivity as a function of GO NP content for GO/collagen samples.

The I-V curves of the pristine collagen and the corresponding composites (Figure 5a) are characterized as a nearly linear regime, in particular for pristine collagen and the samples with lower filler contents. The inclusion of GO NPs into the collagen matrix leads to a slight increase in the slope of the I-V curves due to interfacial effects [40] as well as to an increase of the non-linearity, which indicates an electrically induced activation of mobile species at the interfaces between the filler and polymer [41]. Using Equation (1) and the resistance value obtained from the slope of I-V curves, Figure 5b shows the variation of electrical conductivity with increasing filler content.

Figure 5b shows that the electrical conductivity (*σ*) value increases with increasing GO NP content. For pristine collagen and 0.25 wt % of GO NPs, the *σ* values are 8.7 × 10^−11^ S·cm^−1^ and 3.6 × 10^−10^ S·cm^−1^, respectively, but by increasing the GO NP content up to 3 wt %, a maximum *σ* value of 1.83 × 10^−9^ S·cm^−1^ is obtained. The small increase in the electrical conductivity is mainly attributed to interface effects [40], which provide mobile species for electrical conduction [38,42].

Typically for composites based on GO and natural polymers, the percolation threshold is greater than 10% by weight of GO, where electrical properties depend on charge distribution, interactions between polymer and filler, polymer solvent and processing method, among other factors [43].

### 3.5. Functional Bending Sensor Response

**Figure 6:**
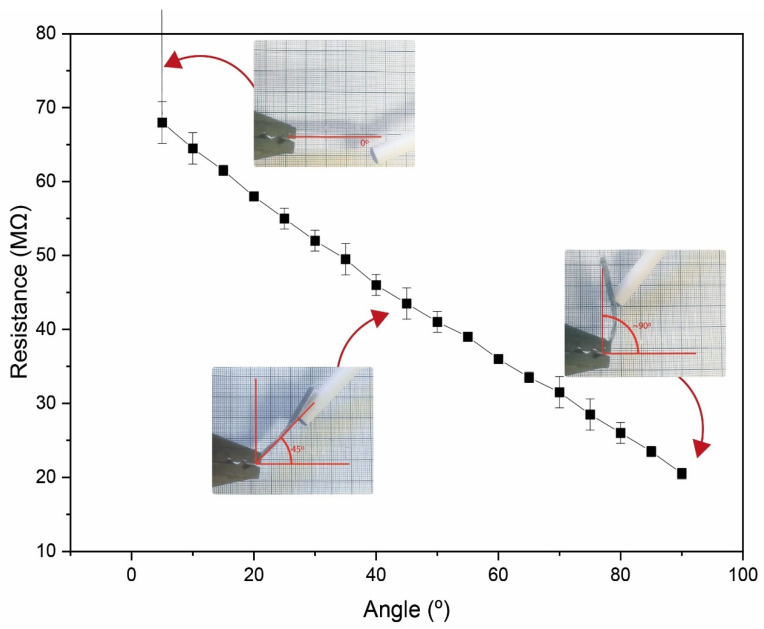
Resistance variation as a function of the bending angle for the GO/collagen sample incorporating 3 wt % of GO NPs.

As the GO/collagen sample with the highest electrical conductivity value was the sample with 3 wt % of GO NPs, this sample was used for a proof of concept regarding the development of a resistive bending sensor (Figure 1a). Figure 6 shows the resistance value variation as a function of the bending angle.

The small air gap creates an initial resistance when the sensor is straight, as the sensor is in an open circuit (Figure 1b and Figure 6). When a bending force is applied, the air gap between the interdigit and the GO/collagen sample is reduced, decreasing the initial electrical resistance. After the GO/collagen film makes contact with the interdigit, the contact surface between the two materials increases, reducing the resistance of the sensor. This resistance variation is inversely proportional to the bending distance, as shown in Figure 6.

**Figure 7:**
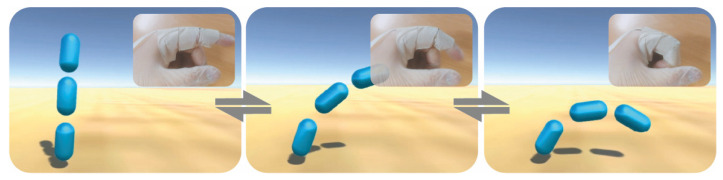
Frames of the developed bending sensor application: movement of the finger and corresponding variation in the computer interface.

As a functional proof of concept, the bending sensor was glued to a glove close to the proximal interphalangeal joint of the index finger, as presented in Figure 7.

In this context, there is a change in resistance when the index finger flexes which is proportional to the flexing angle. A real-time bending application was created on UNITY using the Ardunity library. The application consists of three pill shapes that rotate depending on the value received by the microcontroller. Each increment in the resistance changes the angle in the 3D application, as represented in Figure 7.

Collagen composites with graphene oxide nanoparticles (GO NPs) have been prepared and fully characterized, and functionality has been demonstrated for resistive bending sensors with a focus on the development of sustainable functional materials for advanced applications.

## 4. Conclusions

Collagen composites incorporating different GO NP contents (0, 0.25, 0.50, 0.75, 1 and 3 wt %) were developed by a solvent casting method. Independently of the GO content in the collagen matrix, the thermal stability of all samples increases as a result of the increase in the structural order of collagen, which is related to the GO-collagen physical interactions of hydroxyl and carboxylic groups in GO NPs with amino groups in collagen. Further, all samples present an endothermic peak between 35 °C and 180 °C related to dehydration and the thermal denaturation of the amorphous region of collagen. The presence of GO NPs in the collagen matrix also leads to an increase in the hydrophilic character of the surface as well as to an increase of the tensile strength, from 33.2 ± 2.4 MPa for collagen to 44.1 ± 1.0 MPa for collagen films with 3 wt % GO NPs. The electrical conductivity also increases with the GO content up to a maximum value of 1.83 × 10^−9^ S·cm^−1^ for 3 wt % GO NP content. Additionally, the successful performance of the materials as a resistive bending sensor was demonstrated for collagen film incorporating 3 wt % of GO NPs, decreasing the resistance value proportionally to the increase of the bending angle. Thus, the suitability of the developed natural-based bending sensor for soft robotic applications is demonstrated.

## Figures and Tables

**Table 1:** Glass transition temperature (T_g_), denaturation temperature (T_d_) and enthalpy (ΔH) values obtained by DSC analysis of collagen films containing GO NPs.

Films	T_g_ (°C) ± 1 °C	T_d_ (°C) ± 1 °C	ΔH (mJ/g) ± 1%
Control	46.9	94.2	275.7
0.25GO	48.3	99.4	238.8
0.50GO	48.6	99.1	227.5
0.75GO	48.7	99.4	227.4
1GO	48.9	100.5	219.9
3GO	48.9	101.7	219.7

**Table 2:** Area of the XPS spectra peaks for control and 1GO films.

Area (%)	C–C/C–H 284.6 (eV) ± 0.2 eV	C–O/C–N 286.1 (eV) ± 0.2 eV	C=O/N–C=O 287.5 (eV) ± 0.2 eV	C=N/C–N 399.5 (eV) ± 0.2 eV	O–C=O/O=C–N 531.1 (eV) ± 0.2 eV
**Control**	69.5	3.1	9.3	5.9	12.2
**1GO**	56.0	3.4	14.1	11.7	14.8

**Table 3:** Tensile strength (TS) and elongation at break (EAB) of collagen films prepared with different GO NP contents.

Films	TS (MPa)	EAB (%)
Control	33.2 ± 2.4 ^a^	6.0 ± 0.3 ^a^
0.25GO	35.8 ± 1.3 ^a^	6.0 ± 0.3 ^a^
0.50GO	38.9 ± 2.5 ^a^	6.0 ± 0.3 ^a^
0.75GO	40.2 ± 2.2 ^b^	5.8 ± 0.3 ^a^
1GO	42.0 ± 2.3 ^b,c^	5.5 ± 0.1 ^b^
3GO	44.1 ± 1.0 ^c^	5.4 ± 0.1 ^b^

^a–c^ Two means followed by the same letter in the same column are not significantly (*p* > 0.05) different through the Tukey’s multiple range test.

## Data Availability

Data will be made available on request.
